# A New Dichoptic Training Strategy Leads to Better Cooperation Between the Two Eyes in Amblyopia

**DOI:** 10.3389/fnins.2020.593119

**Published:** 2020-11-26

**Authors:** Zitian Liu, Zidong Chen, Le Gao, Manli Liu, Yiru Huang, Lei Feng, Junpeng Yuan, Daming Deng, Chang-Bing Huang, Minbin Yu

**Affiliations:** ^1^State Key Laboratory of Ophthalmology, Guangdong Provincial Key Laboratory of Ophthalmology and Visual Science, Zhongshan Ophthalmic Center, Sun Yat-sen University, Guangzhou, Guangdong, China; ^2^Key Laboratory of Behavioral Science, Institute of Psychology, Chinese Academy of Sciences (CAS), Beijing, China; ^3^Department of Psychology, University of Chinese Academy of Sciences, Beijing, China

**Keywords:** amblyopia, dichoptic training, dichoptic masking, contrast sensitivity functions, interocular suppression

## Abstract

Recent clinical trials failed to endorse dichoptic training for amblyopia treatment. Here, we proposed an alternative training strategy that focused on reducing signal threshold contrast in the amblyopic eye under a constant and high noise contrast in the fellow eye (HNC), and compared it to a typical dichoptic strategy that aimed at increasing the tolerable noise contrast in the fellow eye (i.e., TNC strategy). We recruited 16 patients with amblyopia and divided them into two groups. Eight patients in Group 1 received the HNC training, while the other eight patients in Group 2 performed the TNC training first (Phase 1) and then crossed over to the HNC training (Phase 2). We measured contrast sensitivity functions (CSFs) separately in the amblyopic and fellow eyes when the untested eye viewed mean luminance (monocularly unmasked) or noise stimuli (dichoptically masked) before and after training at a particular frequency. The area under the log contrast sensitivity function (AULCSF) of masked and unmasked conditions, and dichoptic gain (the ratio of AULCSF of masked to unmasked condition) were calculated for each eye. We found that both dichoptic training paradigms substantially improved masked CSF, dichoptic gain, and visual acuity in the amblyopic eye. As opposed to the TNC paradigm, the HNC training produced stronger effects on masked CSFs, stereoacuity, dichoptic gain, and visual acuity in the amblyopic eye. Interestingly, the second-phase HNC training in Group 2 also induced further improvement in the masked contrast sensitivity and AULCSF in the amblyopic eye. We concluded that the HNC training strategy was more effective than the TNC training paradigm. Future design for dichoptic training should not only focus on increasing the tolerable noise contrast in the fellow eye but should also “nurture” the amblyopic eye under normal binocular viewing conditions and sustained interocular suppression.

## Introduction

Amblyopia, a neurodevelopmental vision disorder caused by abnormal visual experience during early childhood, affects about 2–5% of the population (Kiorpes et al., 1998; Holmes and Clarke, 2006). Amblyopia leads to both monocular deficits in the amblyopic eye, e.g., impaired visual acuity (Levi, 2006), reduced contrast sensitivity (Hess and Howell, 1977; McKee et al., 2003), unsteady monocular fixation (Subramanian et al., 2013), and abnormal binocular vision, e.g., interrupted binocular summation (Huang et al., 2009) and binocular rivalry (Lunghi et al., 2016), asymmetric dichoptic masking (Shooner et al., 2017; Zhou et al., 2018) and interocular suppression (Li J. et al., 2011), and reduced stereoacuity (Levi et al., 2015).

Focusing on improving monocular deficits in the amblyopic eye, patching or penalizing the fellow eye is still the clinical norm in amblyopia treatment (Holmes et al., 2006; Tailor et al., 2016). It is effective at improving visual acuity in the amblyopic eye during early childhood, with efficacy dropped sharply after the age of 7 years (Holmes and Levi, 2018). On the other hand, contrast sensitivity at high frequency, stereoacuity, binocular combination/summation were found to be still deficient in clinically “treated” amblyopia (Zhao et al., 2017), demonstrating the limits of monocular patching and penalization. In the past decades, monocular perceptual learning (PL) of different tasks, e.g., contrast detection with (Polat et al., 2004, 2009; Campana et al., 2014) and without flankers (Zhou et al., 2006; Huang et al., 2008; Hou et al., 2011; Jia et al., 2018), Vernier offset judgment (Levi et al., 1997) and position discrimination (Li R.W. et al., 2005, 2008), was also proposed to recover visual acuity in the amblyopic eye and showed promising results (Levi and Li, 2009; Astle et al., 2011). However, it has been found that monocular training cannot fully normalize binocular vision (Jia et al., 2018). In addition, the improvement of monocular and binocular functions following monocular PL did not correlate with each other, indicating (at least) partially different mechanisms underlying monocular and binocular deficits (Jia et al., 2018). The development of binocular treatment for amblyopia is thus necessary to recover deficient binocular vision in amblyopia.

Emphasizing on the binocular deficits in amblyopia and normalization of interocular balance between the two eyes, several dichoptic training paradigms had been developed to reduce disproportional interocular suppression (Hess et al., 2010; Knox et al., 2012; Li J. et al., 2013b; Ooi et al., 2013; Li J. et al., 2015; Kelly et al., 2018). Using relatively limited number of patients, early well-controlled laboratory-based studies mostly adopted playing video games, such as Tetris (Li J. et al., 2013b) and Dig Rush (Hess et al., 2010; Kelly et al., 2016, 2018), or watching films (Li S.L. et al., 2015; Bossi et al., 2017; Birch et al., 2019), and showed promising results in both children (Birch et al., 2015; Li S.L. et al., 2015; Bossi et al., 2017) and adults (Hess et al., 2010; Li J. et al., 2013b; Ooi et al., 2013; Li J. et al., 2015; Vedamurthy et al., 2015a,b) with amblyopia. However, recent clinical trials that recruited large diversified samples found only mild or modest treatment effect for both children (Holmes et al., 2016; Pediatric Eye Disease Investigator Group, et al., 2019) and adult amblyopia (Gao et al., 2018b), questioned the effectiveness and feasibility of binocular paradigm in recovering both binocular and monocular functions in clinical practice. One possibility is that, with the hope of achieving balanced contribution from the two eyes, typical application usually utilized presentation of low-contrast image, animated pictures, or video to the fellow eye and complimentary high-contrast contents to the amblyopic eye (Hess et al., 2010; Li J. et al., 2013b), which was inherently different from normal binocular viewing condition, in which the incoming stimuli in the amblyopic and fellow eyes are of similar images with the same contrast, and the fellow eye imposes strong and sustained suppression upon the amblyopic eye. Another possibility, e.g., failure to comply and engage continuously, was also proposed (Sloper, 2016), although gaming or film watching was thought to be more enjoyable in the original design.

**FIGURE 1:**
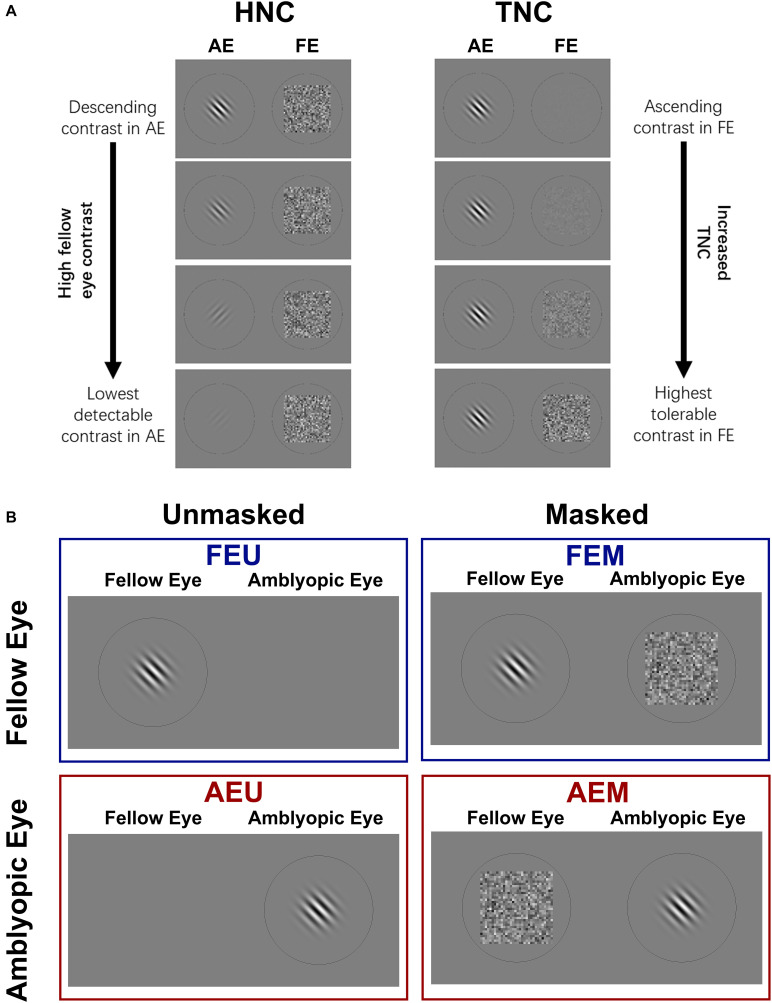
**(A)** Two strategies of dichoptic training used in this study. Left: High noise contrast (HNC) protocol; Sinusoidal gratings were presented to one eye, while a mean background luminance (monocular unmasked condition) or a Gaussian white noise mask (dichoptic masked condition) was presented to the untested eye. From top to bottom: the contrast of gratings in an amblyopic eye (AE) was manipulated from high to low, while noise mask in a fellow eye (FE) was fixed at high contrast (σ = 0.33). With training, the contrast of grating was adjusted (usually decreased) to maintain stable performance. Right: tolerable noise contrast (TNC) protocol; grating contrast in AE was fixed while mask contrast in FE was manipulated from low to high to maintain stable performance. **(B)** Four conditions of CSFs were measured: unmasked fellow eye (FEU), unmasked amblyopic eye (AEU), masked fellow eye (FEM), and masked amblyopic eye (AEM).

Motivated by the well-recognized effectiveness of refractive adaptation in improving monocular visual acuity and reducing interocular suppression (Moseley et al., 2002; Cotter et al., 2006; Gao et al., 2018a; Wang et al., 2018), in which patients were prescribed only appropriate refractive correction, received roughly comparable physical inputs to both eyes, and maintained a high-energy stimulation in the fellow eye, we proposed a new binocular training paradigm that involves presenting high-contrast noise to the fellow eye and gradually decreasing image contrast in the amblyopic eye (or HNC, Figure 1A), aiming to actively “nurture” the amblyopic eye with strong inhibition from the fellow eye. Our HNC approach was related to the monocular contrast detection paradigm, which was found to be effective in improving contrast sensitivity and visual acuity in the amblyopic eye (Zhou et al., 2006; Huang et al., 2008), but prescribed strong and sustained interocular inhibition from the fellow to the amblyopic eye. The HNC paradigm was also related to the dichoptic TNC training paradigm (i.e., tolerable noise contrast; Figure 1B), which was believed to be effective in reducing interocular suppression (Liu and Zhang, 2018, 2019), but differed with it in two aspects: (1) We used contrast detection, a hallmark of spatial vision disorder in amblyopia (Kiorpes et al., 1999), instead of contrast discrimination task, an essentially veridical function in amblyopia (Hess and Bradley, 1980), and (2) We kept noise contrast in the fellow eye constant and adjusted the contrast of the grating in the amblyopic eye, posing a strong suppressive effect to the amblyopic eye and simulating a more typical binocular viewing condition (Li J. et al., 2013a).

By comparing the efficacy of the TNC and HNC strategies in amblyopic patients, we found that the HNC approach not only produced stronger monocular and binocular training effects than the TNC training did but also provided extra visual gains in patients who have previously received the TNC training, demonstrating greater potential in recovering both monocular and binocular deficits in amblyopia.

## Materials and Methods

### Participants

Sixteen amblyopic patients (13 anisometropic, 1 strabismic, and 2 combined) aged 12–32 years (mean ± S.D., 21.1 ± 6.4 years) participated in this study. Participants were recruited from Zhongshan Ophthalmic Center (Guangzhou, China), had no visual training experience, and were all naive to the purpose of the study. Clinical details of all participants are summarized in Table 1.

**TABLE 1:** Clinical details of all participants by group.

Group	Patient	Age, y/sex^*a*^	Eye^*b*^	Refractive error diopter	Visual acuity LogMAR^*c*^	Strabismus^*d*^, prism diopter △	History^*e*^
1	P1	12/M	AE	+4.50/−1.00 × 165°	0.3	None	Detected at 6 y, refractive correction from 6 y, patching for 4 y (2–4 hr/day)
			FE	+1.00	−0.04		
	P2	32/F	AE	+5.75	0.3	None	Detected at 6 y, refractive correction from 6 y, patching for 1 y (2 hr/day)
			FE	+0.75/−0.50 × 90°	0		
	P3	24/F	AE	+4.25/−0.50 × 45°	0.34	None	Detected at 12 y, refractive correction from 12 y, patching for 1 y (2 hr/day)
			FE	−1.50/−0.75 × 90°	0		
	P4	20/M	AE	−8.25/−1.75 × 170°	0.3	None	Detected at 6 y, refractive correction from 6 y, patching for 0.5 y (2 hr/day)
			FE	−3.50/−0.75 × 10°	0		
	P5	20/M	AE	+2.50/−1.50 × 10°	0.5	None	Detected at 6 y, refractive correction from 6 y, patching for 0.5 y (2 hr/day)
			FE	−1.00/−0.75 × 175°	0		
	P6	24/F	AE	−3.00	0.32	ET 5△	Detected at 17 y, used to be ET 40△, refractive correction from 17 y, patching for 0.5y (2 hr/day), surgery at 20 y, ET 5△ now
			FE	−3.00	0		
	P7	16/F	AE	+7.75/−2.00 × 175°	0.86	None	Detected at 8 y, refractive correction from 8 y, patching for 2 y (2 hr/day)
			FE	−3.75/−0.75 × 155°	0		
	P8	16/F	AE	+3.00	0.14	None	Detected at 8 y, refractive correction from 8 y, patching for 2 y (2 hr/day)
			FE	−0.75	−0.08		
2	P9	12/M	AE	+4.75/−1.50 × 160°	0.6	None	Detected at 5 y, refractive correction from 5 y, patching for 3 y (2–4 hr/day)
			FE	+0.75/−0.50 × 175°	−0.04		
	P10	26/F	AE	+9.25/−4.00 × 5°	0.2	ET40△ (Uncorrected) ET 5△ (Corrected)	Detected accommodative esotropia at 6 y, refractive correction from 6 y, patching for 1 y (2–4 hr/day)
			FE	+7.50/−3.00 × 175°	0		
	P11	26/F	AE	+2.75/−1.00 × 15°	0.34	None	Detected at 12 y, refractive correction from 12 y, no patching
			FE	−2.50/−0.50 × 5°	0		
	P12	13/M	AE	+4.25	0.7	None	Detected at 11 y, refractive correction from 11 y, patching for 2 y (4 hr/day)
			FE	+0.25/−0.50 × 180°	−0.06		
	P13	14/F	AE	+6.00/−1.00 × 15°	0.5	ET 5△	Detected at 3 y, used to be ET 35△, refractive correction from 3 y, patching for 0.5 y (2 hr/day), surgery at 12 y, ET 5△ now
			FE	+1.50/−1.00 × 180°	0		
	P14	30/M	AE	+4.75/−1.50 × 180°	0.46	None	Detected at 12 y, refractive correction from 25 y, no patching
			FE	−1.75/−1.25 × 5°	0		
	P15	25/F	AE	+6.25/−1.75 × 135°	0.42	None	Detected at 12 y, refractive correction from 12 y, no patching
			FE	+2.75	0		
	P16	28/M	AE	+4.50/−1.50 × 180°	0.4	None	Detected at 16 y, refractive correction from 16 y, no patching
			FE	−1.50	−0.1		

*^a^M, male; F, female. ^b^AE, amblyopic eye; FE, fellow eye. ^c^LogMAR, the logarithm of the minimum angle of resolution. ^d^△, prism diopter; ET, esotropia. ^e^y, year; hr, hour.*

Inclusion criteria for amblyopic patients were as follows: Patients older than 12 years of age were diagnosed with amblyopia due to a history of anisometropia, strabismus, or both. Amblyopia was defined as an interocular difference in best-corrected visual acuity (BCVA) of 0.2 logMAR or greater (≥2 lines), with a logMAR acuity of at least 0.9 in the amblyopic eye and 0.1 in the fellow eye. Anisometropia was defined as an interocular spherical equivalent difference of 1.50 diopters or more, with or without microtropia. Patients with strabismus were initially diagnosed with esotropia, but were re-aligned with refractive correction and/or surgery to within four prism diopters of orthotropia at near and distance fixations for more than 1 year. Eligible patients had stable visual acuity (no improvement over the most recent three hospital visits) and had worn appropriate glasses for more than 8 weeks, if needed. Patients with combined mechanism had a history of acquired anisometropia and strabismus. Exclusion criteria were inability to cooperate with the eye examinations or psychophysical tests, presence of any coexisting ocular or systemic diseases, congenital infections/malformations, or developmental delay. Three patients were excluded due to the inability to perform the dichoptic training or unstable visual acuity.

### Clinical Measurements

All participants underwent a set of ophthalmologic examinations, including cycloplegic objective and subjective refraction, cover tests at near and distance fixations, slit-lamp and funduscopic examinations. Full refractive correction was provided for all subsequent tests. Visual acuity was measured using a Chinese tumbling E logMAR chart (Mou, 2005; Xi et al., 2014; Jia et al., 2018). Stereopsis was measured using the Random Dot Stereo Acuity Test (Vision Assessment Corp., Elk Grove Village, IL). For patients who were unable to perceive depth at the 500 arcsec, we designated their stereoacuity as 800 arcsec (Liu and Zhang, 2019). All ophthalmologic examination procedures were conducted in the same clinic room under constant lighting conditions.

### Design

The experiment consisted of three phases: pre-training measurement, training, and post-training measurement. In pre- and post-training measurements, visual acuity, different conditions of contrast sensitivity functions (CSFs) for amblyopic and fellow eyes, and stereoacuity were assessed for all patients. The scheme for different conditions of CSF measurement is presented in Figure 1B. We measured each eye’s monocular unmasked CSF in which the untested eye viewed a background with mean luminance, and dichoptically masked CSF in which the untested eye viewed a high-energy noise mask. As a result, four conditions of CSFs were obtained for each patient: fellow eye unmasked (FEU), amblyopic eye unmasked (AEU), fellow eye masked (FEM), and amblyopic eye masked (AEM).

Contrast sensitivity, defined as the reciprocal of contrast threshold, was calculated from sinusoidal grating detection thresholds at spatial frequencies 0.5, 1, 2, 4, 8, and 16 cycles per degree (c/d) for each eye in the unmasked and masked conditions. Since the amblyopic eye is much weaker than the fellow eye and the magnitude of interocular inhibition varied significantly across patients (Chen et al., 2014; Zhou et al., 2018), we measured contrast sensitivity at the masked condition from low to high spatial frequency in sequence and terminated the measurement if contrast sensitivity was below 2.0 (i.e., contrast threshold > 50%) at a particular spatial frequency. As a result, masked CSF in the two eyes was measured with different number of frequencies.

In the training session, we applied and compared the TNC and HNC training strategies in 16 patients, who were randomly divided into two groups. Eight patients in Group 1 performed 8–10 sessions of dichoptic training following the HNC strategy. The other eight patients in Group 2 performed 8–10 sessions of dichoptic TNC training in the first training phase and then crossed over to the HNC training in the second training phase for another 8–10 sessions. Retention of training effects was evaluated in 5 out of 16 patients at 12 months post-training.

### Apparatus and Stimuli

All stimuli were generated and controlled by a PC computer running Matlab (MathWorks, Natick, MA, United States) and Psychtoolbox (version 3.0) (Brainard, 1997; Pelli, 1997). Dichoptic stimuli were rendered on a 3D-ready gamma-corrected computer monitor (ASUS VG278HE; refresh rate: 144 Hz; resolution: 1,920 × 1,080 pixels; background luminance: 54 cd/m^2^), with participants viewing through a pair of polarized glasses (NVIDIA 3D shutter glasses). The viewing distance was 114 cm, and a chin-forehead rest was used to secure the head position. All experiments were conducted in a dimly lit room (<5 lx).

The “signal” stimuli consisted of oriented ( ± 45° from vertical) sinusoidal gratings at six spatial frequencies (0.5, 1, 2, 4, 8, and 16 c/d) with random phase. Each grating consisted of eight cycles. The size of gratings was inversely proportional to the spatial frequency (i.e., 16°, 8°, 4°, 2°, 1°, and 0.5°), keeping the number of cycles the same across different spatial frequencies. Pixel intensities of the random “noise” stimuli were sampled from a Gaussian distribution (μ = 0, σ = 0.33). Noise stimuli in each trial were sampled independently. The size of the noise mask was the same as that of the signal grating (Chen et al., 2014). To minimize edge effects, a half-Gaussian ramp was added around the stimulus.

### Procedure

Before the measurement of CSF and each training sessions, a 0.2° red fixation point and a circle, which was slightly larger than the size of grating, were presented to both eyes in the middle of the screen. Participants were asked to use a computer keyboard to adjust the position of the circles until the two eyes were able to fuse the stimulus. The generated coordinates that marked proper fusion were then used in the subsequent measurement and training.

In a typical trial, an oriented sinusoidal grating and a noise mask or mean luminance background were dichoptically presented for 200 ms to the tested and untested eye, respectively. The orientation was randomly set to be either +45° or −45° from the vertical, with equal probability. Participants were asked to indicate the orientation of the grating by using the “left” or “right” arrow key on the computer keyboard. The red fixation point will change to green for each correct response. The response also initiated the next trial after a 500-ms intertrial interval. Prior to the study, each participant had a chance to practice for 100 trials with high-contrast gratings (80%).

Contrast threshold at each spatial frequency was measured with an adaptive two-down one-up staircase procedure, converging to a performance level of 70.7% correct. Specifically, two consecutive correct responses decreased the grating contrast, and one error raised the grating contrast, and contrast changing from increase to decrease or vice versa was counted as one reversal. Step size of contrast change was 50% (C_n + 1_ = C_n_^∗^ 0.50) before the first reversal and 10% (C_n + 1_ = C_n_^∗^ 0.90) thereafter (Levitt, 1971). Contrast thresholds were calculated as the mean of the last four reversals of the staircase.

### Training

In the dichoptic training phase, grating stimuli were only presented to the amblyopic eye, while the noise stimuli were presented to the fellow eye. Patients in Group 1 performed dichoptic training based on the HNC strategy, in which the amblyopic eye (AE) was trained with a contrast detection task under a high and fixed noise mask (σ = 0.33) in the fellow eye. An adaptive two-down one-up staircase controlled the contrast of the sinusoidal grating in the amblyopic eye upon subject’s judgment and tracked performance of 70.7% correct. Training lasted 8–10 sessions.

Patients in Group 2 first performed dichoptic training based on the TNC strategy, in which the contrast of gratings in AE was fixed at 50% and the contrast energy of noise mask in FE was manipulated by an adaptive two-up one-down staircase that converged to a performance level of 70.7% correct. Specifically, two consecutive correct responses increased the noise level, and one error decreased the noise level, and noise level changing from increase to decrease or vice versa was counted as one reversal. The mean of the last four reversals of the staircase was taken as the maximally tolerable noise level. After the TNC training, patients in Group 2 went through the measurement of visual acuity, stereoacuity, and CSFs. They then received dichoptic HNC training, as described above.

In both Group 1 and Group 2, patients were trained at their individual cut-off spatial frequencies, as determined from the masked CSF measured in the amblyopic eye (average SF in Group 1: 4.00 ± 2.98 c/d; Group 2: 3.75 ± 1.98 c/d; t_14_ = 0.245, P = 0.810) and defined as the spatial frequency at which the contrast threshold under noise mask was 50% (or contrast sensitivity of 2.0).

### Statistical Analysis and Model Fitting

Data are presented as mean ± SEM, unless otherwise specified. Learning curve (i.e., log_1__0_ contrast sensitivity as a function of log [training session]) was fitted with a linear function (Zhou et al., 2006; Huang et al., 2008):

$$\log_{10}C\,S\,\left(s\,e\,s\,s\,i\,o\,n\right)=C\,S_{0}+\alpha \times \log_{10}\left(s\,e\,s\,s\,i\,o\,n\right),$$

where *CS* denotes contrast sensitivity at a particular session, *CS*_0_ is the intercept, and α is the slope of the learning curve (learning rate, or unit improvement at the trained condition). When analyzing CSF in the amblyopic eye, only spatial frequencies with measurable contrast sensitivity (contrast threshold < 100%) in most patients were included (0.5–8 c/d for unmasked condition, 0.5–2 c/d for masked condition), while CSF across all spatial frequencies (0.5–16 c/d) was analyzed for the fellow eye.

The average CSF of each group measured before and after training was fitted by a parabolic function in log–log scale (Huang et al., 2008; Chen et al., 2016; Zhou et al., 2018). An overall contrast sensitivity metric, the area under the log contrast sensitivity function (AULCSF), was determined by calculating the definite integration of the best-fitted function from 0.5 c/d to the cut-off spatial frequency (Lesmes et al., 2010; Chen et al., 2016). To index the effect of dichoptic masking on CSF, or dichoptic gain (Shooner et al., 2017), we calculated the ratio of AULCSF of masked to unmasked CSF, varying from 0 (completely masked by the other untested eye) to 1 (no masking effect from the other eye).

For each patient, the magnitude of improvements (Zhou et al., 2006) for each measure (e.g., AULCSF, dichoptic gain magnitude, visual acuity, and stereoacuity) was defined as:

(1) $$I\,m\,p\,r\,o\,v\,e\,m\,e\,n\,t=\frac{\mathrm{Post}\,\_\,\mathrm{training}\,\mathrm{Measure}-\mathrm{Pre}\,\_\,\mathrm{training}\,\mathrm{Measure}}{\mathrm{Post}\,\_\,\mathrm{training}\,\mathrm{Measure}}\times 100\%\;$$

Data from pre-training and post-training measurements were compared using a two-tailed paired *t*-test. The magnitude of improvements for the two groups was compared using a two-tailed independent sample *t*-test.

To evaluate the retention effect of training, we calculated the retention coefficient (Zhou et al., 2006) of each measure (e.g., AULCSF, dichoptic gain, visual acuity, and stereoacuity) as:

(2) $$R\,e\,t\,e\,n\,t\,i\,o\,n\,C\,o\,e\,f\,f\,i\,c\,i\,e\,n\,t=\frac{\text{Retested}\,\text{Measure}-\mathrm{Pre}\,\_\,\mathrm{training}\,\text{Measure}}{\mathrm{Post}\,\_\,\mathrm{training}\,\text{Measure}-\mathrm{Pre}\,\_\,\mathrm{training}\,\text{Measure}}\times 100\%\;$$

## Results

### Outcomes of HNC Protocol Training (Group 1)

**FIGURE 2:**
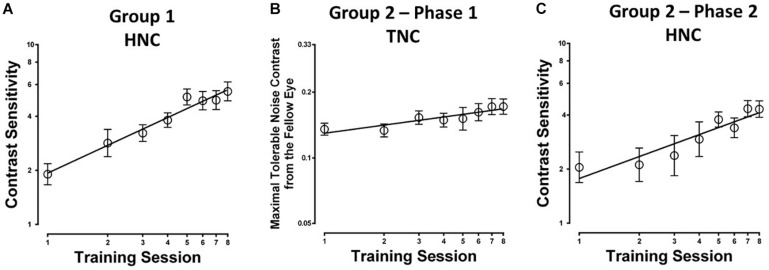
Learning curves for the two different training protocols in amblyopic participants from **(A)** Group 1 with constant and high noise contrast in the fellow eye (HNC), **(B)** Group 2 (Phase 1) with progressively elevated noise contrast in the fellow eye (TNC); **(C)** Group 2 (Phase 2), HNC training.

Eight to ten sessions of contrast detection training in the amblyopic eye under constant and high-energy noise mask from the fellow eye led to significant improvement in contrast sensitivity at the trained spatial frequency (254.51 ± 64.08%; *t*_7_ = 5.100, *P* = 0.001; calculated from pre- and post-training masked CSF measurement). The average learning curve (i.e., contrast sensitivity as a function of training sessions) is shown in Figure 2A. The HNC training improved contrast sensitivity with a slope of 1.55 log units per log training session (*R*^2^ = 0.97, *P* < 0.01).

**FIGURE 3:**
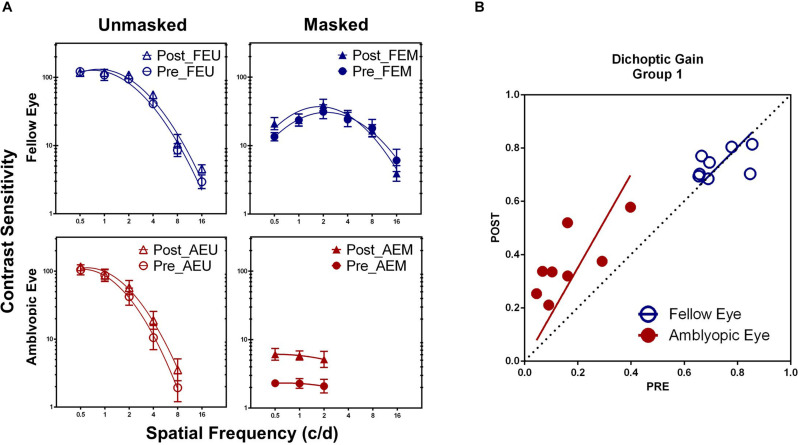
**(A)** Pre- and post-training contrast sensitivity functions (CSFs) under different conditions in Group 1. **(B)** Dichoptic gains for the two eyes before HNC training (x-axis) are plotted against those after training (y-axis). The diagonal unity line represents unchanged dichoptic gain between pre- and post-training assessment.

Training at one spatial frequency also facilitated contrast sensitivity at other untrained frequencies in the amblyopic eye, if tested under high masking from the fellow eye. Treating practice level (pre-/post- training) and spatial frequency (0.5, 1, and 2 c/d) as within-subject factors, a repeated-measure analysis of variance (ANOVA) showed that the AEM contrast sensitivity varied significantly with practice level [*F*_(1_, _7__)_ = 31.411, *P* = 0.001, η^2^ = 0.818], but not with spatial frequency [*F*_(2, 14)_ = 0.429, *P* = 0.660, η^2^ = 0.058] and interaction of the two factors [*F*_(2, 14)_ = 0.013, *P* = 0.987, η^2^ = 0.002], indicating a general contrast sensitivity improvement. CSF also showed significant change with practice level [*F*_(1, 7)_ = 10.436, *P* = 0.014, η^2^ = 0.599], spatial frequency [0.5–8 c/d, *F*_(4, 28)_ = 155.321, *P* < 0.001, η^2^ = 0.957], and interaction of the two factors [*F*_(4, 28)_ = 3.005, *P* = 0.035, η^2^ = 0.300] in the amblyopic eye, if tested with mean background in the fellow eye (AEU condition; Figure 3A). On the contrary, CSF in the fellow eye did not change significantly with practice, either at the FEU condition [*F*_(1, 7)_ = 3.615, *P* = 0.099, η^2^ = 0.341] or the FEM condition [*F*_(1, 7)_ = 0.439, *P* = 0.529, η^2^ = 0.059].

Averaged across patients, AULCSF improved about 266.21 ± 66.04% (*t*_7_ = 7.066, *P* < 0.001) in the AEM condition and 13.32 ± 4.01% (*t*_7_ = 3.370, *P* = 0.012) in the AEU condition. The AULCSF improvement showed significant difference between the two conditions (*t*_7_ = 3.871, *P* = 0.006) but without correlation (*r* = 0.206, *P* = 0.624), which implied at least partially different mechanisms underlying the improvement of AEM and AEU contrast sensitivity. For the fellow eye, the AULCSF improvement in neither the FEM (*t*_7_ = 1.303, *P* = 0.234) nor the FEU (*t*_7_ = 2.092, *P* = 0.075) condition reached significance, indicating eye-specific learning.

To estimate the effects of HNC training on interocular interaction in amblyopia, we calculated and compared the magnitude of dichoptic gain before and after training. After HNC training, dichoptic gain improved in the amblyopic eye by 222.28 ± 56.71% (*t*_7_ = 6.546, *P* < 0.001), but remained unchanged in the fellow eye (2.16 ± 3.48%, *t*_7_ = 0.372, *P* = 0.721, Figure 3B). In other words, the masked CSF improved more than the unmasked CSF did in the amblyopic eye, demonstrating the increased strength of the amblyopic eye in counteracting the inhibition from the fellow eye.

After the HNC training, visual acuity in the amblyopic eye improved by 0.83 ± 0.14 lines (*t*_7_ = 5.954, *P* = 0.001), while that of the fellow eye remained unchanged (*t*_7_ = 0.798, *P* = 0.451). The HNC training also improved stereoacuity from 362.50″ ± 82.24″ to 272.00″ ± 88.84″ (*t*_7_ = 2.695, *P* = 0.031). Interestingly, two out of three stereoblind patients (unable to identify geometric shapes at the largest disparity of 500 arcsecs) obtained measurable stereopsis after HNC training (to 500″ and 400″, respectively). The improvement of visual acuity in the amblyopic eye, dichoptic gain, and stereoacuity did not correlate with each other (all *P* > 0.10).

### Outcomes of TNC Protocol Training (Group 2; Phase 1)

Patients in Group 2 first performed TNC training for 8–10 sessions (Phase 1), in which signal contrast in the amblyopic eye was fixed and noise in the fellow eye was progressively elevated (Liu and Zhang, 2018, 2019), and then underwent HNC training for an additional 8–10 sessions (Phase 2) to test if HNC training could bring extra benefits.

The average learning curve (i.e., the maximal tolerable noise contrast in the fellow eye as a function of training sessions) for Phase 1 is shown in Figure 2B. TNC training increased maximal tolerable noise contrast in the fellow eye with a slope of 0.013 units per training session (*R*^2^ = 0.83, *P* = 0.002). The maximal tolerable noise contrast significantly elevated by 48.9 ± 12.5% (*t*_7_ = 7.408, *P* < 0.001; calculated from the measurement at the first and last training sessions).

**FIGURE 4:**
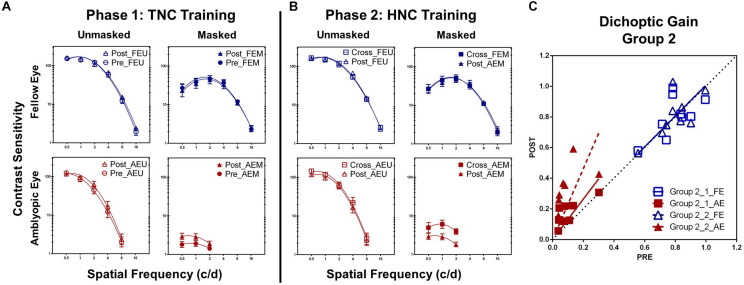
**(A)** CSFs under different conditions in Group 2 before (Pre, circle) and after (Post, triangle) the TNC training (Phase 1). **(B)** CSFs under different conditions in Group 2 after the TNC training (Post, triangle) and after crossed over to the HNC training (Cross, square) (Phase 2). **(C)** Dichoptic gains for the two eyes in Group 2 before training (*x*-axis) is plotted against those after training (*y*-axis) for the two phases. The diagonal unity line represents unchanged dichoptic gain between pre- and post-training assessment.

After TNC training, AEM contrast sensitivity at the training spatial frequency was significantly improved by 60.30 ± 26.19% (*t*_7_ = 4.364, *P* = 0.003; calculated from pre- and post-training masked CSF). Contrast sensitivity at other untrained frequencies in the amblyopic eye also elevated significantly, if tested under high masking from the fellow eye (AEM condition). ANOVA revealed that AEM contrast sensitivity varied significantly with practice level [*F*_(1, 7)_ = 7.924, *P* = 0.026, η^2^ = 0.531] and spatial frequency [*F*_(2, 14)_ = 6.363, *P* = 0.011, η^2^ = 0.476] but not with interaction of the two factors [*F*_(2, 14)_ = 0.442, *P* = 0.651, η^2^ = 0.059, Figure 4A]. On the other hand, CSF did not change significantly following training at all other conditions [AEU condition, *F*_(1, 7)_ = 4.921, *P* = 0.062, η^2^ = 0.413; FEU condition, *F*_(1, 7)_ = 2.299, *P* = 0.173, η^2^ = 0.247; FEM condition, *F*_(1, 7)_ = 1.328, *P* = 0.287, η^2^ = 0.159].

Averaged across patients, the AULCSF of the AEM condition significantly improved by 97.52 ± 26.82% (*t*_7_ = 3.948, *P* = 0.006), whereas the AULCSFs of the other conditions showed no significant changes (AEU, 7.89 ± 4.08%, *t*_7_ = 2.296, *P* = 0.055; FEU, 2.88 ± 1.76%, *t*_7_ = 1.529, *P* = 0.170; FEM, 6.57 ± 3.13%, *t*_7_ = 1.097, *P* = 0.309). For dichoptic gain, we also found significant change in the amblyopic eye (82.20 ± 23.03%; *t*_7_ = 3.666, *P* = 0.008) instead of the fellow eye (3.66 ± 4.86%; *t*_7_ = 0.643, *P* = 0.540, Figure 4C).

TNC training improved amblyopic visual acuity by 0.34 ± 0.13 lines (*t*_7_ = 2.664, *P* = 0.032), while that of the fellow eye remained unchanged (*t*_7_ = 1.001, *P* = 0.351). However, we found no significant changes in stereoacuity after TNC training (*t*_7_ = 1.000, *P* = 0.351). No significant correlation was found among the three measures (all *P* > 0.10).

### Additional Benefits of HNC Training in Patients With TNC Training History (Group 2; Phase 2)

**FIGURE 5:**
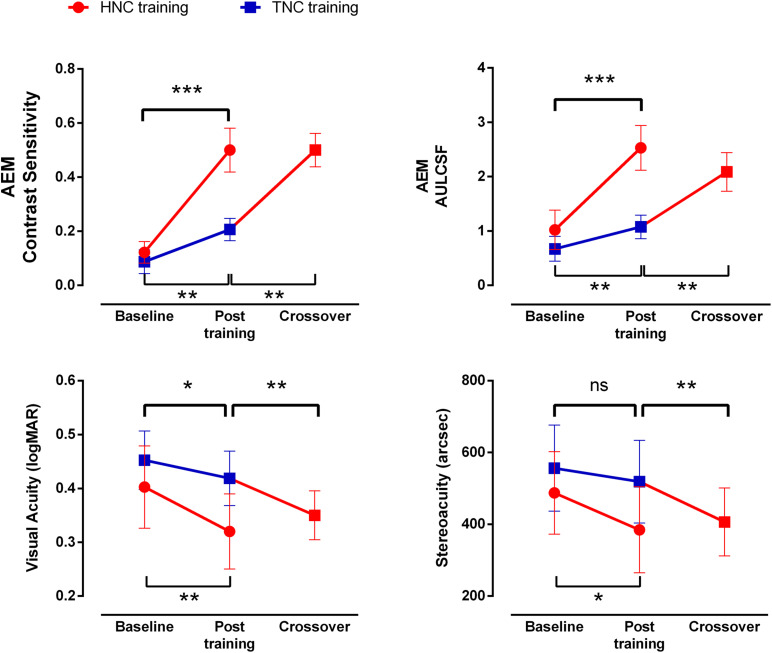
Improvements in visual function after HNC vs. TNC training. Error bars represent SEM. *p < 0.05; **p < 0.01; ***p < 0.001.

After the second phase of HNC training, AEM contrast sensitivity got a further improvement of 179.84 ± 31.88% (*t*_7_ = 10.076, *P* < 0.001; calculated from pre- and post-training masked CSF, Figure 5) at the training spatial frequency, with a slope of 1.22 log units per log training session (*R*^2^ = 0.87, *P* = 0.001, Figure 2C).

A within-subject analysis of variance (ANOVA) showed that AEM contrast sensitivity varied significantly with practice level [*F*_(1, 7)_ = 10.914, *P* = 0.016, η^2^ = 0.609], but not with spatial frequency [*F*_(2, 14)_ = 3.472, *P* = 0.065, η^2^ = 0.332; Figure 4B]. Interaction between the two factors was also not significant [*F*_(2, 14)_ = 0.681, *P* = 0.525, η^2^ = 0.089]. In contrast, there was no significant changes in the AEU condition [*F*_(1,7)_ = 0.816, *P* = 0.369, η^2^ = 0.104], FEU condition [*F*_(1, 7)_ = 0.291, *P* = 0.606, η^2^ = 0.040] and FEM condition [*F*_(1, 7)_ = 0.078, *P* = 0.788, η^2^ = 0.011].

Patients in Group 2 gained additional improvement in AULCSF of AEM condition (113.99 ± 26.60%, *t*_7_ = 4.333, *P* = 0.003, Figure 5), but not in the AEU condition (2.19 ± 3.05%, *t*_7_ = 2.070, *P* = 0.077) and the fellow eye conditions (FEU: *t*_7_ = 0.887, *P* = 0.405; FEM: *t*_7_ = 0.157, *P* = 0.880). A further improvement was found in dichoptic gain in the amblyopic eye (105.63 ± 25.25%, *t*_7_ = 3.987, *P* = 0.005), instead of the fellow eye (*t*_7_ = 0.190, *P* = 0.855, Figure 4C).

After HNC training in Phase 2, a significant further improvement in visual acuity of 0.69 ± 0.18 lines was observed in the amblyopic eye (*t*_7_ = 3.847, *P* = 0.006, Figure 5), but not in the fellow eye (*t*_7_ = 0.357, *P* = 0.732). There was also significant improvement in stereoacuity (from 418.75″ ± 81.25″ to 368.75″ ± 78.45″; *t*_7_ = 2.646, *P* = 0.033, Figure 5). Similarly, there was no significant correlation among the acuity improvement in the amblyopic eye, the improvement in stereoacuity, and the improvement of dichoptic gain in the amblyopic eye (all *P* > 0.10).

### Comparison of Training Strategies

**FIGURE 6:**
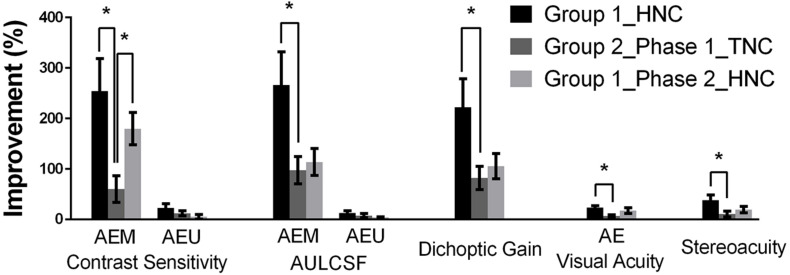
Summary of training effects. Groups were represented by different colors: black (Group 1), dark gray (Group 2, Phase 1), and light gray (Group 2, Phase 2). Error bars represent SEM. *p < 0.05.

By comparing the data from Group 1 and the first training phase of Group 2, we evaluated the efficacies of the two training strategies (HNC vs. TNC) on recovering monocular and binocular performance in amblyopia, e.g., the magnitudes of improvement, measured in terms of percent change of AULCSF at AEM, AEU, dichoptic gain, visual acuity, and stereoacuity (Figure 6).

For the amblyopic eye, we found that the HNC training produced significantly greater improvements in the AEM contrast sensitivity (*t*_14_ = 2.841, *P* = 0.025) at the training spatial frequency, AEM AULCSF (*t*_14_ = 2.367, *P* = 0.033) and dichoptic gain (*t*_14_ = 2.289, *P* = 0.038), indicating that the HNC strategy is more effective than the TNC strategy in promoting the amblyopic eye to counteract the masking effect from the fellow eye. On the contrary, no significant difference between the two strategies was found in the magnitude of improvements of AEU AULCSF (*t*_14_ = 0.947, *P* = 0.359) and AEU contrast sensitivity (*t*_14_ = 0.118, *P* = 0.908) at the training spatial frequency. Both training strategies produced no significant change in FEU and FEM CSFs in the fellow eye.

The HNC training strategy also produced significantly greater improvement in visual acuity in the amblyopic eye (*t*_14_ = 2.670, *P* = 0.018) and stereoacuity (*t*_14_ = 2.998, *P* = 0.010) than the TNC strategy did. Both training strategies produced no significant change in visual acuity of the fellow eye.

### Retention

Retention of training effects was evaluated for five patients (two patients from Group 1, the other three from Group 2) 12 months after training. The AEM contrast sensitivity and AEM AULCSF were almost fully retained, with mean retention coefficients of 90.1 ± 49.3 and 106.90 ± 14.52% (mean ± SEM). The AEU contrast sensitivity at cut-off spatial frequency and AEU AULCSF were also retained, with mean retention coefficients of 79.4 ± 34.2 and 93.65 ± 16.78%, respectively. The dichoptic gain for the amblyopic eye was also well retained, with a mean retention coefficient of 106.14 ± 14.29%. The average retention coefficient of improvement on visual acuity and stereoacuity was 82.28 ± 23.70 and 110.0 ± 22.4%. Overall, the training effects of both dichoptic training paradigms were robust.

## Discussion

In the current study, we proposed a new binocular training paradigm (i.e., HNC) and compared the efficacy of HNC and TNC protocols in adults with amblyopia. Although both strategies triggered significant learning, the HNC strategy produced more improvements in dichoptically masked CSF, dichoptic gain, visual acuity, and stereoacuity in the amblyopic eye. Furthermore, patients who had been trained with TNC strategy gained additional benefits in monocular and binocular performances from extra phase of HNC training.

Unlike recent dichoptic training paradigms that usually penalized the fellow eye to construct an artificial environment of equal contribution from two eyes in performing particular tasks, e.g., playing a video game and/or watching a film (Hess et al., 2010; Knox et al., 2012; Li J. et al., 2013b, Li J. et al., 2015; Li S.L. et al., 2015; Birch et al., 2015; Vedamurthy et al., 2015a,b; Kelly et al., 2016, 2018; Bossi et al., 2017; Birch et al., 2019), refractive correction provided roughly comparable physical inputs to both eyes and maintained a high-energy stimulation in the fellow eye. In other words, refractive adaptation could be considered as a binocular treatment approach with the presence of stronger inhibition from the fellow to the amblyopic eye, but in a more passive way (Wang et al., 2018). Several studies showed that refractive correction alone successfully improved the amblyopic visual acuity in both children (Moseley et al., 2002; Cotter et al., 2006) and some adults (Gao et al., 2018a). A recent psychophysical study reported that refractive adaptation also reduced interocular suppression, suggesting that the corrected amblyopic eye gradually acquired enhanced competence in binocular vision, even with constant and high suppression from the fellow eye (Wang et al., 2018). In the current study, we developed a new dichoptic training paradigm (e.g., HNC) that maintained high-contrast noise stimuli in the fellow eye, aiming to practice the amblyopic eye in a more intensified condition. Since all our subjects wore appropriate glasses for at least 8 weeks before they participated in the experiment, our results demonstrated the extra benefits of active dichoptic training with sustained high suppression from the fellow to the amblyopic eye. Further improvements in both monocular and binocular functions after the second-phase HNC training in Group 2 also confirmed the efficacy of the HNC protocol.

We found that the magnitude of visual improvement, especially in binocular performance, following the new HNC training strategy was larger than the TNC strategy. Moreover, we observed pronounced further improvement in dichoptic masked CSF, dichoptic gain, visual acuity in the amblyopic eye, as well as stereoacuity in patients of Group 2 who had prior training experience with TNC. These results implied that although both strategies could help the amblyopic eye to exclude the masking effect from the fellow eye, training under a high and constant noise contrast (HNC) was probably more effective than training with progressive noise contrast (TNC) in the fellow eye. Gratings with gradually decreased contrast in the amblyopic eye and noise with high contrast in the fellow eye may better simulate the normal binocular viewing condition, in which the fellow eye exerts constant and stronger inhibition over the amblyopic eye (Huang et al., 2009, 2011). Our HNC approach may have encouraged the amblyopic eye to cooperate more actively with the fellow eye (Mitchell et al., 2003; Murphy et al., 2015).

Our results about TNC training differed from those of Zhang et al. in terms of improvement in stereoacuity, who found a significant (410.9″ ± 70.7″ to 152.7″ ± 35.9″) improvement in stereoacuity (Liu and Zhang, 2019). Although both studies exploited a similar strategy, the intensity of training was different, with more sessions of training (17 sessions) performed in Zhang et al., likely leading to larger visual improvements (Li R.W. et al., 2008). In addition, all our patients had received refractive correction and fellow eye patching therapy for more than 2 months, while most of the patients in the study of Zhang et al. were naive to clinical treatment, especially refractive correction (Gao et al., 2018a). Moreover, the extent of anisometropia was relatively larger (4.59 vs. 3.82 diopters), and the initial amblyopic visual acuity was better (0.45 vs. 0.63 logMAR) in our study, which may also contribute to the discrepancy in visual improvements (Zhou et al., 2006; Dobson et al., 2008).

Patients in Group 1 showed a significant but small improvement in monocular unmasked contrast sensitivity, while those in Group 2 showed no improvement. These results suggest that unlike monocular training (Zhou et al., 2006; Huang et al., 2008; Chen et al., 2016; Jia et al., 2018), dichoptic training might not relieve the intrinsic limitations of the amblyopic eye (Hess et al., 1978; Levi and Klein, 1985; Levi et al., 1999; Xu et al., 2006; Huang et al., 2007, 2009). One possibility is that relatively low training spatial frequency was used in our study (2–4 c/d), which has been proved to be less effective than high spatial frequency in recovering monocular functions (Zhou et al., 2006). Moreover, training under noise and clear display may involve asymmetric transfer characteristics, e.g., training at clear display can benefit performance in noise display but not vice versa, indicating different underlying mechanisms (Dosher and Lu, 2005; Xie and Yu, 2019). On the other hand, there was no significant correlation between all improvements in binocular and monocular vision after HNC training, similar to a previous study (Jia et al., 2018). Zhang et al. found that monocular training further reduced the contrast threshold of the amblyopic eye of patients with dichoptic TNC training experience (Liu and Zhang, 2019). Taken together, these results suggested that dichoptic and monocular training have (at least partially) different mechanisms of improving amblyopic vision. Joint application of monocular and dichoptic training protocols has great potential in amblyopia treatment and is worthy of future investigation (Levi and Li, 2009; Xi et al., 2014; Levi et al., 2015; Jia et al., 2018).

One may argue that our findings were related to the enhanced adaptation in the fellow eye due to continuous presentation of high-contrast noise image in it (Gardner et al., 2005; Kohn, 2007). We do not think it is possible. First, learning effects were found be more prominent with masked condition, indicating of specific learning effects. Second, we performed a retest on five patients 12 months post-training and found robust retention of the training effects (e.g., visual acuity and monocular unmasked CSF) following HNC training. On the contrary, our results suggested that dichoptic and monocular training may involve different mechanisms (e.g., bias the top–down attention toward the amblyopic eye) (Liu and Zhang, 2019). Another limitation for this study is the lack of a reversed crossover, i.e., HNC training followed by TNC training, without which we could not determine the exact interaction of the two training strategies. Our results, on the other hand, provided evidence that HNC training could bring extra benefits after intensive TNC training. We did not find significant difference in improvement after HNC training in Group 1 and after both TNC and HNC training in Group 2, in terms of visual acuity (*t*_14_ = 0.741, *P* = 0.483), stereopsis (*t*_14_ = 0.682, *P* = 0.544), AEM contrast sensitivity at the trained spatial frequency (*t*_14_ = 0.632, *P* = 0.548), and AEM AULCSF (*t*_14_ = 0.410, *P* = 0.694), lending further support to the existence of extra benefits from HNC training, as opposed to TNC training.

To our knowledge, this is the first study that made direct comparison between two possible strategies of dichoptic training in amblyopia. Our results favored the strategy with constant high-contrast noise in the fellow eye and progressive contrast reduction in the amblyopic eye, which might be different in nature from the dichoptic training paradigm that aimed to increase maximal tolerable noise in the fellow eye and better represented the normal binocular viewing conditions.

## Data Availability Statement

The datasets generated for this study are available on request to the corresponding author.

## Ethics Statement

The studies involving human participants were reviewed and approved by the institutional review board of Zhongshan Ophthalmic Center, Sun Yat-sen University. Written informed consent to participate in this study was provided by the participants’ legal guardian/next of kin, for the publication of any potentially identifiable images or data included in this article.

## Author Contributions

ZL, ZC, C-BH, and MY designed the study, contributed to the writing of the manuscript, analyzed the data, and prepared the figures. ZL, ZC, ML, and JY prepared the experiment. ZL, ZC, LG, YH, and LF tested the participants. All authors contributed to the article and approved the submitted version.

## Conflict of Interest

The authors declare that the research was conducted in the absence of any commercial or financial relationships that could be construed as a potential conflict of interest.

## Abbreviations

HNC, high noise contrast: referred to the new dichoptic training strategy with high-contrast noise in the fellow eye in this study
TNC, tolerable noise contrast: referred to the typical dichoptic training strategy aimed at increasing the tolerable noise contrast in the fellow eye
CSFs: contrast sensitivity functions
AULCSF: the area under the log contrast sensitivity function
FEU, fellow eye unmasked: CSF of the fellow eye with no mask but background luminance in the amblyopic eye
AEU, amblyopic eye unmasked: CSF of the amblyopic eye with no mask but background luminance in the fellow eye
FEM, fellow eye masked: CSF of the fellow eye with masks in the amblyopic eye
AEM, amblyopic eye masked: CSF of the amblyopic eye with masks in the fellow eye.
